# A US/Mexico Study of Joint Associations of Physical Activity and Sedentary Behavior on Anthropometric Indicators, Migration Status, Country of Birth and Country of Residence

**DOI:** 10.3390/ijerph15061283

**Published:** 2018-06-17

**Authors:** Katia Gallegos-Carrillo, Britni R. Belcher, Genevieve F. Dunton, Jonathan M. Samet, Lourdes Baezconde-Garbanati

**Affiliations:** 1 Epidemiology and Health Services Research Unit, Instituto Mexicano del Seguro Social, Cuernavaca, Morelos 62000, México; 2 Department of Preventive Medicine, Keck School of Medicine, University of Southern California, Los Angeles, CA 90032, USA; bbelcher@usc.edu (B.R.B.); dunton@usc.edu (G.F.D.); baezcond@usc.edu (L.B.-G.); 3 Colorado School of Public Health, University of Colorado, Aurora, CO 80045, USA; jon.samet@ucdenver.edu

**Keywords:** acculturation, obesity, sedentary, physical activity, migration, nativity

## Abstract

Background: This study examined the influence of migration status, nativity and country of residence on joint associations of physical activity (PA) and sedentary behavior (SB) in anthropometric indicators of Mexicans and Mexican-Americans living in the US and in Mexico. Methods: We examined data from two large national surveys, the National Health and Nutrition Examination Survey from the US (NHANES, 2011–2012) and Mexico (ENSANUT, 2012). Using self-reported minutes of moderate to vigorous physical activity and SB, we calculated four categories for analyses. Anthropometric measures consisted of body mass index (BMI) and waist circumference (WC). We used data of migration status, nativity and country of residence. Linear regression models examined how joint categories of PA and SB were associated with BMI and WC according to migration status, nativity and country of residence, controlling for health risk behaviors. Results: Analyses showed that even among those in the category with the lowest risk behavior, “physically active and low sedentary”, there were differences in BMI and WC by migration status, nativity and country of residence. Within this lower risk category, Mexican immigrants living in the US had the greatest association with high BMI, while US-born Mexican-Americans living in the US had the highest WC values when compared with the group of Mexicans living in Mexico. Conclusions: Joint categories of PA and SB were associated with BMI and WC by migration status, nativity and country of residence among populations with Mexican ethnicity.

## 1. Introduction

Several hypotheses on migration have emerged to explain the ways in which exposure to a new society influences the health of immigrant populations [1,2] Among immigrants to the United States, evidence has suggested that Latinos have better health behaviors such as physical activity and consumption of a diet high in vegetables, fruits and legumes, which are at times found to be healthier than those of non-Latino whites [3,4]. Yet, among some ethnic groups that migrate to the US, there is evidence of increased risk for certain health conditions, due to new and potentially less healthy behaviors they are exposed to upon arrival [5,6]. This is also the case of obesity rates among Mexican-Americans (body mass index ≥ 30). Mexico has the second highest prevalence of obesity worldwide (32.4%) [7] after the US (38.2%) [8], yet Mexican-Americans living in the US have the highest prevalence of obesity at 45.9% [9]. Interestingly, evidence suggests that in addition to country of residence and country of birth, migration status is also a variable in the risk of obesity; Mexican immigrants living in the US have a lower average BMI and rates of obesity than US-born Mexican-Americans [10]. However, it is not yet clear what other factors are contributing to differences in populations that likely share a similar genetic susceptibility for obesity. There is some evidence to suggest that some contributing factors include cultural values [11], stress linked to migration [12,13] and socioeconomic status [14], but also to environmental and epi-genetic factors [15] and exposure to an obesogenic environment. This obesogenic environment includes changes in dietary behavior [16,17], different food patterns (less home meal preparation and higher reliance on fast and convenience food) [18], less physical activity [19] and recent evidence about additional health risks from excessive sitting and time spent in sedentary activities [20].

Further consideration is needed of the role of physical activity (PA) and sedentary behaviors (SB) as contributors to the obesity epidemic. Evidence from studies using US national databases, measuring PA objectively, have observed that the quantity and type of PA differ by ethnicity and migratory status in the US [6], suggesting that PA tends to change when migrants move from lower-medium income countries to high-income countries. Mexican-Americans (including US-born and Mexican immigrants) are the largest group of immigrants in the US (at 63%) and make up 10% of the United States population. In some areas of the US, the percentage of individuals of Mexican origin is even higher. [21]. Previous studies have suggested that US-born Mexican-Americans spend more minutes per week in leisure time PA, but less minutes of PA at work and during commuting, which presents a different pattern of PA than their Mexico-born counterparts living in the US [19]. 

Considering the various studies on SB and PA, relatively few have assessed the interactions between them [22], and those that have reported inconclusive results [23,24]. The evidence is even scarcer for differences among groups with diverse backgrounds, including ethnicity and migration status. This has important implications, as large longitudinal studies have shown that leisure time PA does not always counteract the negative health effects of SB overall [20,25,26,27]. Further, these studies did not include populations that account for different contextual and environmental factors that are presented by migration.

Even, methodologically, the constructs of SB and PA have not always been clearly defined and have sometimes been used interchangeably when they are actually measuring different things. For example, the term “insufficiently active” implies not reaching PA US recommended standards, while “sedentary behavior” denotes low-energy-expenditure activities like sitting, using a computer or personal devices, watching television, sitting time during commuting and remaining in a reclining posture for an extended period of time [28]. Using these definitions, an individual may have both high levels of sedentary behavior and low levels of moderate-to-vigorous physical activity levels (MVPA) at the same time; as these are not independent of each other [29]. Multi-country studies have shown that low SB and PA levels increase metabolic [30] and cardio-metabolic risk [31,32,33,34]. However, to our knowledge, the combination of PA and SB on the anthropometric indicators of a population with different migratory status, country of residence and country of birth has not been previously studied. 

The purpose of this study was to use two large national databases from the US and Mexico to examine if anthropometric measures of Mexican immigrants living in the US, US-born Mexican-Americans living in the US and Mexicans living in Mexico vary according to combined categories of SB and PA in each respective national sample.

## 2. Materials and Methods

We examined data from the 2011–2012 National Health and Nutrition Examination Survey (NHANES) and the 2012 National Health and Nutrition Survey of Mexico (ENSANUT, initials in Spanish). Both the NHANES in the US and ENSANUT from Mexico are parallel cross-sectional studies. They contain similar types of behavioral self-report questionnaires and share the goal of assessing the health status and nutrition of children and adults in each respective country (US and Mexico). 

### 2.1. Design and Characteristics of the Population Study

#### 2.1.1. National Health and Nutrition Examination Survey

NHANES is a four-stage, national area probability survey with fixed sample-size targets for sampling domains, which are defined by race and Hispanic ethnic origin, sex, age and income status. To meet the domain specifications, race and Hispanic origin designations were made. The Hispanic category includes all Hispanic persons regardless of any other self-identified race (such as Caucasian, or white) [35]. During 2011 and 2012, a multistage stratified probability cluster design was used to obtain a nationally-representative sample of the civilian, non-institutionalized population residing in the 50 states of the US and the District of Columbia. [35].

Data collection for the NHANES sample includes a household screener to determine if any household members are eligible for the study [36]. During 2011–2012, there were approximately 11,500-screened households per year. Data were collected from 13,431 individuals. Of those selected 9756 completed the interview. Data for men and women ages 20–69 years, self-identified as “Mexican-Americans”, were included in this study. Of those who self-identified as Mexican-American, there were missing data for the following: SB (n = 505) or outlier values (n = 2), body mass index (BMI) (n = 25), acculturation variables (n = 2) and/or other covariates (n = 17). In addition, the data of pregnant women (n = 6) and participants with underweight BMI (< 18.5 kg/m^2^) (n = 4) were excluded from the analysis. Pregnant women were excluded from the analysis because their BMI increases in a short period of time. We used data from 449 participants with complete information for the variables of interest. 

#### 2.1.2. National Health and Nutrition Examination Survey from Mexico

ENSANUT was conducted between October 2011 and May 2012, using a probabilistic multistage stratified sample and a clustered design. The sample size was representative of all of Mexico (both rural and urban). The data were multiplied by sampling weights or expansion factors in order to get data representative of the national level [37]. ENSANUT collected data from 50,528 households to obtain a sample of 89,000 individuals in Mexico. The information for this study in particular included socio-demographic and PA data obtained from 10,729 adults, representing a population of 65,252,418 individuals. The ENSANUT methodology has been published elsewhere [37].

From ENSANUT, we considered men and women between 20 and 69 years of age for our analyses. Those with missing sedentary behavior data (n = 365), BMI (n = 427) and other covariates (n = 90) and data of underweight participants (< 18.5 kg/m^2^) (n = 99) and pregnant women (n = 226) were excluded, for the reasons explained previously. For this study, our final number was 9702 participants with complete data. 

### 2.2. Measurement

#### 2.2.1. Anthropometrics Measurements

NHANES [38] and ENSANUT [39] both used standardized procedures and trained interviewers to measure height, weight and waist circumference during the physical examination. BMI values were obtained by calculating weight (kilograms) divided by the square of the height (meters). Waist circumference was measured in standing position and was measured just above the uppermost lateral border of the right ilium. Both variables were measured and analyzed as continuous variables.

#### 2.2.2. Physical Activity Levels and Sedentary Behavior

SB was defined as time spent sitting, time watching TV and other forms of screen time (computer use and electronic personal devices). PA included minutes per week of moderate and vigorous physical activity (including walking). Previously-validated scales of physical activity were used in the NHANES and ENSANUT. In the NHANES (2011–2012), this information was collected with the Global Health Activity Questionnaire developed by the World Health Organization [40], while in the ENSANUT 2012, the Spanish version of the International Physical Activity Questionnaire (IPAQ), in its short form, was used [41]. 

In the NHANES survey, PA information was based on responses obtained from the Global Physical Activity Questionnaire (GPAQ) items. The PA measurement included minutes of moderate and vigorous PA during leisure time, activities at work and while traveling to and from places in a typical week, in bouts of at least 10 minutes [42]. The MVPA per week variable was constructed by multiplying minutes of PA of vigorous intensity by two and then adding minutes of moderate physical activity per week. The sedentary behavior (SB) was measured with one question that included sitting time in a typical day, considering different domains of possible SB (at work, leisure time and entertainment and while commuting). SB was measured using minutes per day of sitting time [40].

In the ENSANUT survey, PA information was based on responses obtained from the IPAQ items. The PA measurement included minutes of moderate (including walking) and vigorous physical activity. Questions were asked about frequency and intensity of MVPA in three domains: leisure time, at work and during commuting from one place to another in the last seven days that lasted in bouts of at least 10 minutes. MVPA per week was calculated in the same way as the NHANES database for each participant. The validity and reliability of the IPAQ-Short Form from Mexico has been well documented [43]. Measurement of SB in the ENSANUT survey was through two questions: one that asked about time spent sitting at work, home and leisure time and one about the time spent sitting on transportation in a typical day. This information was aggregated to get minutes per day of SB. The physical activity and sedentary behavior variables in the two databases were cleaned and processed following established protocols [40,41] (IPAQ guidelines, GPAQ Analysis guide). Outlier values of 16 hours or more were not considered in this analysis [40].

#### 2.2.3. Categories of Physical Activity and Sedentary Behavior

According to the recommendation of the World Health Organization physical activity guidelines [44], we used total minutes per week spent in MVPA to classify each participant into one of two categories: as “physically inactive” if the cut-off point of MVPA was < 150 minutes/week and “physically active” if MVPA values were > 150 minutes/week. SB in minutes per week was processed as a two-category variable. However, due to the lack of international guidelines about sedentary cut-off points that could be useful in both the US and Mexico [45], we utilized the median observed in each population as the cut-off point. Individuals were classified as having “low sedentary behavior” if the values of SB were below the median; while individuals with values above the median were classified as having “high sedentary behavior”. 

Based on these classifications of MVPA and SB variables, we constructed four categories of PA and SB, which were mutually exclusive. We followed a behavioral approach from lower to higher risk for the following categories: (a) physically active and low sedentary (most active), (b) physically active and high sedentary (active), (c) physically inactive and low sedentary (moderately active) and (d) physically inactive and high sedentary (least active).

#### 2.2.4. Measurement of Migration Status, Country of Birth and Country of Residence 

The information from both the NHANES and ENSANUT databases was used to stratify the participants in this study based on data of migration status, country of birth, country of residence and ethnicity. All ENSANUT participants were Mexican-born and living in Mexico. Participants in NHANES were sub-classified as either “born in Mexico and living in the US (i.e., Mexican immigrants living in the US)” or “Mexican-Americans-born and living in the US”. Considering this information, the categories considered for the analyses were: (a) Mexican-born living in Mexico, (b) Mexican immigrants living in the US and (c) US-born Mexican-Americans living in the US

#### 2.2.5. Covariates

Information about socio-demographic characteristics were considered covariates, including age, gender and education. Education was categorized at 3 levels: elementary or less, less than high school and high school or above. Employment status considered two categories: employed and unemployed. Health risk conditions were assessed by self-report. The participant was considered a smoker if they responded positively to the question: “Have you smoked at least 100 cigarettes in your life?” Those who reported having been previously given the diagnosis of type II diabetes by a health professional were labeled diabetic. Diagnosis by a health professional was also used to determine whether the individual had high blood pressure, or hypertension, and a high cholesterol level.

### 2.3. Statistical Analyses

Due to the complex sampling of each study, we used a cluster and strata design. Sampling weights were considered in weighted descriptive analyses, including means, ranges and proportions. These were used to describe the characteristics of the study population stratified by each strata of acculturation. We calculated the weighted values (N; % and confidence intervals) of the three populations set up by the data of migration status, country of birth and country of residence in each exclusive category of PA and SB. 

In a second stage of analysis, we combined the national databases from the US and Mexico and analyzed them together to assess if the migration status, country of birth and country of residence influenced BMI and WC. We conducted separate multivariate linear regression models for BMI and WC, for each one of the 4 exclusive MVPA and SB categories. The variable combining migration status, country of birth and country of residence was considered the independent variable. The group “Mexico-born living in Mexico” was the reference group. We carried out separate regression models considering both non-adjusted and adjusted analyses. The adjusted model included the socio-demographic variables and health risk conditions as potential confounders (tobacco use, diabetes, high blood pressure and high levels of cholesterol) of the study population simultaneously. All regression analyses considered a *p*-value of < 0.05 to be statistically significant. 

## 3. Results

### 3.1. Sociodemographic Characteristics and Categories of Physical Activity and Sedentary Behavior

Table 1 presents the characteristics of the study population, combining migration status, nativity and country of residence data. The “most active” category represented the greatest proportion of the sample: 47% of Mexico-born living in Mexico, 33.5% of Mexican immigrants living in the US and almost 56% of those US-born Mexican-Americans living in the US The “moderately active” category represented the lowest proportion of the sample (only 4.6% of the US-born Mexican-Americans living in the US). However, in the group of Mexican immigrants living in the US, the “least active category” was also the highest; almost 30% compared to other groups. 

Those who were classified in the category of “moderately active” had the highest proportion of diabetes and high blood pressure (22.8% and 49.3% of US-born Mexican-Americans living in the US, respectively). Mexico-born individuals living in Mexico who were also classified as “moderately active” showed the highest rate of unemployment (58.6%). The top level of education (high school graduate and higher) was observed among US-born Mexican-Americans living in the US (80% were high school graduates or higher in the category of “active”). Among the group of Mexico-born living in Mexico, about one third of them were classified in the “least active” category. The Mexico-born living in Mexico in this joint category of PA and SB had higher levels of education (38.3%) in comparison to the other joint categories of PA and SB. 

**Figure 1:**
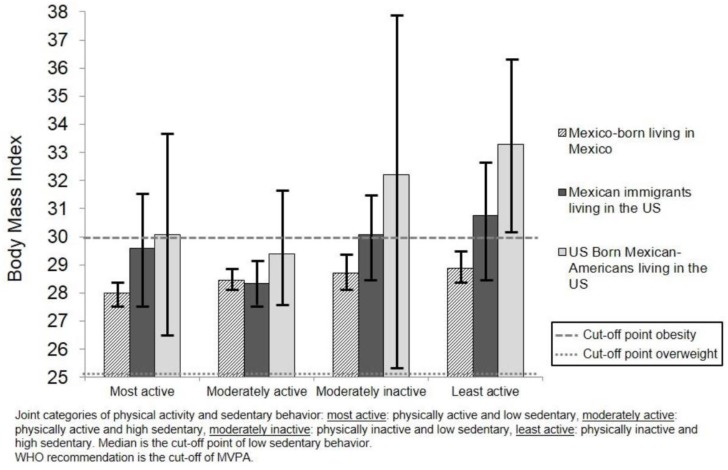
Mean of body mass index among Mexican-born living in Mexico and Mexican immigrants and Mexican-Americans living in the US according to joint categories of moderate-to-vigorous physical activity (MVPA) and sedentary behavior.

**Figure 2:**
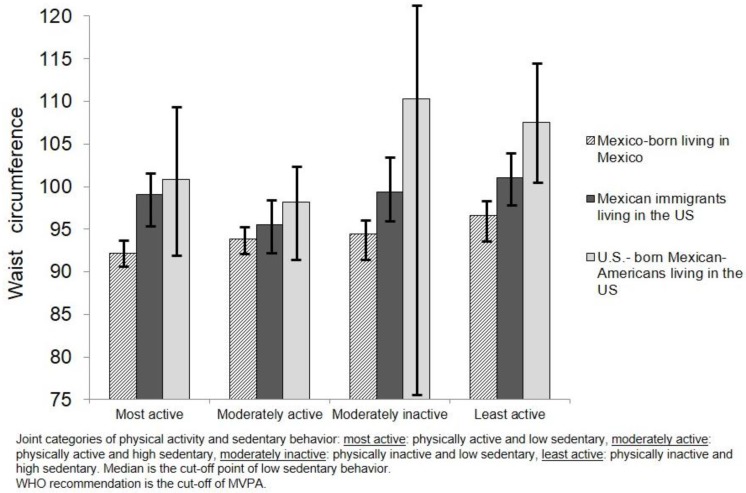
Mean of waist circumference among Mexican-born living in Mexico and Mexican immigrants and Mexican-Americans living in the US according to joint categories of physical activity and sedentary behavior.

Analyses assessing BMI and WC showed that even among those in the category with the lowest risk behaviors, there were observed differences in BMI and WC by migration status, birthplace and country of residence; US-born Mexican-Americans living in the US had the highest values of BMI and WC in comparison with the other two groups (Figure 1 and Figure 2). 

### 3.2. Associations between Categories of Physical Activity and Sedentary Behavior on BMI and Waist Circumference by Migration Status, Country of Birth and Country of Residence

Table 2 presents the unadjusted and adjusted analysis of the association between joint categories of PA and SB on BMI levels and WC according to migration status, country of birth and country of residence (Mexico-born living in Mexico as the reference group). 

The results on BMI and WC are presented considering those with lower risk as “most active” (physically active and low sedentary) and those with higher risk as “least active” (physically inactive and high sedentary). The results in the category “most active” showed that being a Mexican immigrant living in the US was associated with a 1.32-kg/m^2^ greater BMI (β = 1.32, 95% C.I. 0.1, 2.7) when compared to those who were Mexico-born living in Mexico. These associations remained significant when adjusting for socio-demographic factors and health risk conditions (*p* < 0.05). Within the category of “moderately inactive”, being a US-born Mexican-American living in the US was associated with higher BMI values (β = 3.1, 95% C.I. 0.5, 6.8), which remained significant in adjusted models (*p* < 0.05). This pattern was also observed in the category with the highest risk of “least active.” US-born Mexican-Americans living in the US had a higher BMI (β = 4.6, 95% C.I. 2.8, 6.4) when compared to Mexico-born individuals still living in Mexico. These findings were also statistically significant in both unadjusted and adjusted models for socio-demographic variables and health risk conditions (*p* < 0.001). 

The results showing the association between categories of PA and SB on WC considering the migration status, country of birth and country of residence are also presented in Table 2 (Mexico-born living in Mexico as the reference group). In the lowest risk behavior of “most active”, the Mexican immigrants living in the US (β = 6.85, 95% C.I. 1.5, 12.1) and the US-born Mexican-Americans living in the US (β = 11.3, 95% C.I. 2.9, 19.5) showed a greater association with higher WC values compared to those born and still living in Mexico. However, after adjusting for covariates and potential confounders, this finding was no longer statistically significant. This trend was also observed in the “moderately active” category. Only US-born Mexican-Americans living in the US showed a significant association with higher WC values (β = 7.65, 95% C.I. 3.2, 12) (*p* < 0.001). 

Those in the riskier group “least active” had higher WC values in comparison to groups with more PA active and less SB, but US-born Mexican-Americans living in the US had the highest WC values (β = 15.3, 95% C.I. 7.6, 23.1) compared to Mexico-born individuals living in Mexico. Mexican immigrants living in the US showed increased values in WC in both categories of “moderately inactive” (β = 11, 95% C.I. 1.35, 20.7) and “least active” (β = 9.6, 95% C.I. 3.2, 15.9). However, these associations were not significant in the adjusted models. 

## 4. Discussion

This analysis explored the association within four distinct categories of PA and SB on anthropometric measures of ethnically Mexican populations living in Mexico and the US According to our results, migration status, country of birth and country of residence influenced BMI and WC values independently of PA and SB. This same effect was observed in other studies. This further suggests that PA alone may not be enough to counteract the negative health implications of sedentary behaviors [20,25,26,27]. We observed that the migration process influenced anthropometric indicators in such a way that the Mexican-Americans that were born and living in the US had higher anthropometric levels than their counterparts that were born in Mexico and living in the US.

The impact of the migration process and the exposure to new environmental factors and cultural values on the health status of Mexican-Americans and particularly on PA [46,47], physical inactivity during leisure time [48] and SB has been previously investigated using the NHANES databases and with data from other studies [19]. Yet, this is the first time that the data have been compared to behaviors observed in a reference group that consists of a population from which the immigrant group originated. To our knowledge, this study is the first of its kind to combine the categories of PA and SB and their associations with anthropometric markers, among those with Mexican ethnicity with varied migratory status and in regard to country of birth. 

This study is consistent with previous studies that have examined associations using national databases and objective measurement of both PA and SB [49], as well as their association with cardio-metabolic markers [34,50]; other studies found similar results, but our study is unique in that we combined the categories of PA and SB using data from populations with shared ethnicity. In the case of obesity, this has important implications, as the genetic component is an important influence of the predisposition to weight gain and obesity [51]. In this case, the results suggest that the exposure of migration changes socio-economic and cultural contexts, environmental factors and therefore influences levels of PA and SB [20,27,45]. In general, past studies have seen that those who are both less sedentary and spend more time engaging in MVPA have better health outcomes, among them lower BMI and WC [34]. 

However, when examining migratory populations, there is scant and inconclusive evidence on the associations between PA and SB. As observed in previous studies, we would assume the less physically active and sedentary the population, the greater their BMI and WC levels. However, those individuals who were most active and born in Mexico (living either in the US or Mexico) had higher values of BMI and waist circumference than those who were moderately active. Consistent with other studies, it appears that PA did not mitigate the negative impact of being sedentary [21,43]. However, also, it seems that there are other contextual factors at play [3], not only migration status, but country of birth, as well. In a study on the Texas-Mexico border, Sharkey et al. [16] found a higher consumption of fast-food and sugar-sweetened beverages among US-born women than those born in Mexico. This suggests that among Mexican-Americans born in the US, BMI and WC could have increased gradually given that they had been less physically active and more sedentary. However, those born in Mexico who had migrated to the US that were similarly inactive and sedentary did not have increased levels. These differences require further in-depth research not available with the information from this study. 

In addition, these findings demonstrate that there are differences in PA and SB due to working conditions, transportation and available leisure time that could be affected by country of birth [6,19]. The contextual factors (migratory status, country of birth and country of residence) that influence the balance between PA and SB and other health behaviors that we observed in this study make individuals more prone to the negative effects of a sedentary lifestyle; as was observed in the anthropometric values of the population of this study. These results remained significant in the models even after adjusting for sociodemographic variables and health risk among Mexican-Americans. An additional explanation about lower BMI and WC among Mexican-immigrants living in the US is the influence of selective migration [52]. Under this assumption, the migrant population is positively selected to both the labor market and health status. Being in better physical shape to begin with allows them to get jobs that require higher physical effort and greater energy expenditure, including related to PA when at work; which is likely associated with a lower BMI. Longitudinal studies of nationally-representative samples, focused on recent migration from Mexico to the United States, showed weak evidence that supports the assumption that the Mexican migrant population is healthier than their counterparts born and living in the US [53].

It is possible that there was overestimation of PA levels due to the self-reporting nature of the data. In this study, the minutes of sedentary activity and MVPA per day in each of the combined categories show relative consistency with the findings reported previously by other research works [34]. In our study, Mexican individuals born and living in Mexico, US-born Mexican-Americans and Mexican immigrants living in the US, in the category with the higher risk behavior (“least active”), reported an average of five minutes of MVPA and 433.4 minutes (seven hours) of sedentary behavior per day. In contrast, in a health survey from England, in which PA and SB were measured with accelerometers, a mean of 9.7 minutes of MVPA and 596 minutes (around nine hours) of sedentary behavior per day was seen [34]. However, it is important to mention that the comparability between these studies is limited; measures of physical activity, for example, were different for each study.

The results of this study showed associations among a combination of categories of PA and SB according to migration status, country of birth and country of residence. Among those (US-born Mexican-Americans living in the US), the category “moderately active” was the most prevalent; with 56% of the participants falling into this pattern. Among participants who were born in Mexico, this category was also the most prevalent, although the proportion was lower (46.6% of Mexican immigrants living in the US and 33.5% Mexico-born living in their country of origin, i.e., Mexico). Almost 30% of the Mexican immigrants living in the US were classified as “least active.” This could mean that societal and economic barriers (such as fewer hours working, poor housing conditions, limitations in accessing recreational and fitness facilities and challenging conditions related to immigration) are impacting healthy behaviors. Our data were consistent with what has been shown previously [48,54,55]. 

### Limitations

The varying measures in binational studies in the US and Mexico for analyzing joint associations of PA and SB through different questionnaires limit the ability to get an accurate estimation of the outcome variables and the magnitude of the associations. Further, we are aware of the limitations of using self-report measures such as the GPAQ [40] in the NHANES and the IPAQ [41] in the ENSANUT, to assess PA and SB, due the biases associated [56] with self-report measures. However, our results are consistent with other studies that have estimated the impact of acculturation on the levels of SB and PA among Mexican-Americans and Mexicans living in the US [19,48]. These studies have contributed to the evidence about the impact of physical activity and sedentary behaviors in the context of the acculturation process to the US of foreign-born Mexican-Americans, in ways that had not been done previously. 

We recognize that in previous studies of the NHANES, measurements of PA [51] and sedentarism [26] had fewer limitations, as they were collected through accelerometry [49,50]. However, using these data was not possible, because there has been no accelerometry measurement for the ENSANUT in Mexico. Once there are more comprehensive measurements of PA and SB during working hours, transportation time and leisure time, we can better estimate the impact of acculturation on health outcomes among immigrants from Mexico to the US Additionally, there is a lack of consensus among international guidelines regarding recommendations on sedentary behaviors [45]. To our knowledge, there are no international guidelines established that present an accepted and adequate cut-off point for defining sedentary behaviors. For this reason, we proposed a conservative approach to define sedentarism from participants and created categories of high and low SB using the mean observed in the study population. This is consistent with definitions used in similar studies and allows for comparability among studies [34]. Despite this binational study using national health surveys, the information about the diagnosis of health conditions has been measured through self-report from the participants about previous medical diagnoses. Therefore, health risk conditions such as diabetes, high blood pressure and high cholesterol could be underestimated, particularly among Mexican immigrants, as previous studies have found that immigrants, mainly the unauthorized immigrants, had a lower access to healthcare, including preventative services, hospitalization [57,58] and even lower access to the emergency department [59]. 

## 5. Conclusions

Even in a context in which a population is adapting to a new culture, being more physically active is not necessarily ameliorating the negative impact of the sedentary behavior. Additionally, being of Mexican ethnicity and having been born in the US were associated with higher BMI and WC independent of categories of physical activity and sedentary behavior. Future research should identify the potential causes of these associations. Further research can also explore the same population based on data from the time when they lived in Mexico and following them through the life course experiences as they immigrate and adjust to life in the United States. This paper has implications for the development of intervention programs that can target specific behaviors in specific groups in both Mexico and the United States. Further research that examines these variables within the context of reception by the US is also warranted. 

## Figures and Tables

**Table 1:** Characteristics of the population with Mexican ethnicity according to joint categories of physical activity and sedentary behavior and migration status, birth place and country of residence. National Health and Nutrition Databases from the United States and Mexico (NHANES 2011–2012 and ENSANUT 2012).

Characteristic	Mexico-Born Living in Mexico	Mexican Immigrants Living in the US	US-Born Mexican-Americans Living in the US	Mexico-Born Living in Mexico	Mexican Immigrants Living in the US	US-Born Mexican-Americans Living in the US
	Most active			Moderately active		
	(N = 21,272,889) (n = 3944)	(N = 2,104,861) (n = 67)	(N = 889,926) (n = 26)	(N = 26,469,663) (n = 4127)	(N = 2,919,679) (n = 88)	(N = 3,399,165) (n = 91)
	% (C.I. 95%)	% (C.I. 95%)	% (C.I. 95%)	% (C.I. 95%)	% (C.I. 95%)	% (C.I. 95%)
Age (in years) *	41.3 (40.4, 42.1)	39 (35, 43)	39.6 (32.5, 46.6)	38.5 (37.7, 39.4)	39.4 (36.3, 42.6)	34.3 (31.9, 36.7)
**Sex** % (95% C.I.)						
Male	41.5 (38.6, 44.4)	53.9 (38.6, 69.1)	66.7 (38.2, 95.3)	49.4 (46.6, 52.2)	62.9 (49.7, 76.1)	53.9 (42.4, 65.4)
Female	58.4 (55.5, 61.3)	46.1 (30.8, 61.4)	33.2 (4.7, 61.8)	50.5 (47.7, 53.3)	37.1 (23.9, 50.2)	46.1 (34.6, 57.6)
**Education level** % (95% C.I.)						
Less than high school	83.8 (81.6, 86)	67.9 (46.9, 88.8)	35.2 (−6.01, 76.5)	63.8 (60.7, 66.9)	55.8 (43.2, 68.4)	19.1 (12.4, 25.8)
High school graduate and more	16.2 (13.9, 18.3)	32.1 (11.1, 53)	64.7 (23.49, 106)	36.1 (33.1, 39.2)	44.2 (31.5, 56.7)	80.1 (74.2, 87.6)
**Occupation** % (95% C.I.)						
Employment	53.1 (50.1, 56.1)	75.6 (61.6, 98.7)	64.5 (32.8, 96.2)	57.4 (54.3, 60.5)	76.4 (64.2, 83)	70.7 (60.4, 80.9)
Unemployment	46.8 (43.8, 49.8)	24.3 (10.2, 38.4)	35.5 (3.8, 67.2)	42.6 (39.5, 45.7)	26.4 (16.9, 35.7)	29.3 (19.1, 39.6)
**Health risk conditions** % (95% C.I.)						
Diabetes	8.4 (6.6, 10)	10.3 (0.001, 20.7)	13.7 (−0.72, 28.2)	7.4 (6, 8.8)	7.9 (8.5, 15.1)	3.5 (−2.2, 9.3)
High blood pressure	14.7 (12.7, 16.8)	15.2 (3.9, 26.4)	36.1 (−1.2, 73.4)	13.6 (11.6, 15.7)	13.9 (6.3, 21.5)	21.8 (10.6, 33.1)
High cholesterol	12.7 (10.5, 14.8)	19 (7.6, 30.3)	29.2 (9.3, 49.2)	13.7 (11.8, 15.7)	20 (12.4, 27.6)	18.7 (7.4, 29.9)
**MVPA** (minutes/day) *	159.4 (153.2, 165.5)	196.8 (136.1, 257.5)	190.3 (105.2, 275.3)	147.1 (140.8, 153.5)	178.4 (133.2, 223.5)	152.7 (121, 184.5)
**Sedentary time** (minutes/day) *	117.3 (114.3, 120.3)	84.5 (70.5, 98.5)	96.2 (77.2, 115.3)	378.8 (368.6, 389)	268. (1 (239.4, 296.9)	355 (300.2, 411.4)
	Moderately inactive			Least active		
	(N = 3,410,936) (n = 707)	(N = 1,220,304) (n = 39)	(N = 278,715) (n = 11)	(N = 5,684,364) (n = 924)	(N = 2,475,596) (n = 81)	(N = 1,505,528) (n = 46)
Age (in years) *	41.5 (40, 42.9)	40.9 (36.2, 45.6)	47.3 ( −11.9, 106.5)	40.1 (38.5, 41.7)	40.3 (38.1, 42.5)	37.6 (32.5, 42.6)
**Sex** % (95% C.I.)						
Male	33.5 (27.3, 39.6)	38.4 (16.3, 60.5)	31.4 (−1.5, 2.1)	50.1 (44, 56.2)	48.2 (31.7, 64.7)	34.2 (22.4, 46)
Female	66.4 (60.3, 72.6)	61.5 (39.5, 83.7)	68.6 (−1.1, 2.5)	49.8 (43.7, 55.9)	51.8 (35.3, 68.3)	65.8 (53.9, 77.6)
**Education level** % (95% C.I.)						
Less than high school	82.5 (77.8, 87.2)	83.1 (66.7, 99.3)	34.7 (−2.44, 3.14)	61.7 (56.1, 67.3)	65.6 (54.4, 76.9)	31 (8.7, 53.3)
High school graduate and more	17.4 (12.7, 22.1)	16.9 (6.1, 33.2)	65.2 (−2.14, 3.44)	38.3 (32.7, 43.8)	34.3 (23, 45.6)	68.9 (46.6, 91.2)
**Occupation** % (95% C.I.)						
Employment	41.3 (35.4, 47.3)	54 (32.7, 75.3)	46.1 (−3.26, 4.19)	60.2 (54.7, 65.7)	63.3 (42.8, 83.8)	70.1 (47.7, 92.5)
Unemployment	58.6 (52.6, 64.6)	45.9 (24.7, 67.2)	53.9 (−3.19, 4.26)	39.8 (34.2, 45.3)	36.7 (16.2, 57.2)	29.9 (7.5, 52.3)
**Health risk conditions** % (95% C.I.)						
Diabetes	10.6 (7, 14.3)	11.8 (−0.9, 24.4)	22.8 (−2.05, 2.51)	13.7 (9.8, 17.6)	10.4 (1.37, 19.37)	9.4 (5.8, 13.1)
High blood pressure	15.4 (11.6, 19.2)	21.5 (4.8, 38.1)	49.3 (−1.29, 2.27)	15.5 (12.3, 18.8)	18.7 (6.4, 31)	22.8 (7.4, 38.2)
High cholesterol	13.6 (9.5, 17.6)	14.9 (2.1, 27.7)	13.2 (−54.7, 81.2)	13.5 (10.1, 16.9)	25.4 (17.2, 33.6)	20.7 (8.1, 33.3)
**MVPA** (minutes/ day) *	8.2 (7.3, 9.1)	3.8 (1.27, 6.4)	7.3 (−27.6, 42.3)	7.8 (7.1, 8.6)	4.7 (3.1, 6.3)	4.3 (1.1, 7.49)
**Sedentary time** (minutes/day) *	107.3 (100.4, 114.2)	87.7 (67.8, 107.6)	81.7 (−99.8, 263.3)	481.1 (437.6, 524.7)	334 (284.8, 383.2)	485.2 (386, 584.4)

* Mean: MVPA, moderate-to-vigorous physical activity (self-reported). Joint categories of physical activity and sedentary behavior: most active: physically active and low sedentary, moderately active: physically active and high sedentary, moderately inactive: physically inactive and low sedentary, least active: physically inactive and high sedentary.

**Table 2:** Joint associations of physical activity and sedentary behavior on the body mass index and waist circumference of Mexico-born living in Mexico, Mexican immigrants and Mexican-Americans living in the US National Health and Nutrition Databases from the United States and Mexico (NHANES 2011–2012 and ENSANUT 2012).

Joint Categories of Physical Activity and Sedentary Behavior	Body Mass Index				Waist Circumference			
	Mexican Immigrants Living in the US		US-Born Mexican-Americans Living in the US		Mexican Immigrants Living in the US		US-Born Mexican-Americans Living in the US	
	Non-adjusted ¥	Adjusted §	Non-adjusted ¥	Adjusted §	Non-adjusted ¥	Adjusted §	Non-adjusted ¥	Adjusted §
	β (95% C.I.)	β (95% C.I.)	β (95% C.I.)	β (95% C.I.)	β (95% C.I.)	β (95% C.I.)	β (95% C.I.)	β (95% C.I.)
Most active	1.32 (0.1, 2.7) *	1.3 (0.02, 2.6) *	1.5 (−0.5, 3.6)	1.28 (−0.73, 3.3)	6.85 (1.5, 12.1) **	4.54 (−0.5, 9.5)	11.3 (2.9, 19.5) **	6.85 (−1.1, 14.7)
Moderately active	−0.54 (−1.7, 0.6)	−0.43(−1.5, 0.69)	0.64 (−0.5, 1.8)	0.88 (−0.2, 1.9)	3.72 (−0.8, 8.3)	0.8 (−3.5, 5.1)	7.65 (3.2, 12.1) **	6.86 (2.6, 11.1) **
Moderately inactive	0.57 (−1.4, 2.5)	0.02 (−1.8, 1.9)	3.4 (0.05,6.8)	3.1 (−0.1, 6.4)	11 (1.35, 20.7) *	5.76 (−3, 14.5)	12.3 (−4.4, 29)	5.3 (−10.1, 20.7)
Least active	1.26 (−0.1, 2.7)	1.01 (−0.4, 2.3)	4.6 (2.8, 6.4) **	4.1 (2.4, 5.8) **	9.6 (3.2, 15.9) *	5.6 (−0.3, 11.5)	15.3 (7.6, 23.1) *	14.4 (7.2, 21.7) *

Reference category: Mexico-born living in Mexico. ¥ Standardized regression coefficients (β) indicating the change in standard units of the dependent variable (joint categories of physical activity and sedentary) due to the increase in the standard unit of each variable. § Standardized regression coefficients (β) indicating the change in standard unit of the dependent variable due to the increase in the standard unit of the independent variable. Joint categories of physical activity and sedentary behavior: most active: physically active and low sedentary, moderately active: physically active and high sedentary, moderately inactive: physically inactive and low sedentary, least active: physically inactive and high sedentary. Coefficients (β) in the adjusted model (§) adjusted by the following variables: age, educational level, occupation and health risk conditions (tobacco use, diabetes, high blood pressure, high cholesterol). * *p* < 0.05, ** *p* > 0.0001.
